# Vitamin K as an Endocrine Modulator: Mechanistic Links to Glucose Metabolism and Beyond

**DOI:** 10.3390/nu18081183

**Published:** 2026-04-09

**Authors:** Wojciech Matuszewski, Mikołaj Madeksza, Michał Szklarz, Aleksandra Rutkiewicz, Joanna Rutkowska, Joanna Maria Harazny

**Affiliations:** 1 Clinic of Endocrinology and Metabolic Diseases, School of Medicine, Collegium Medicum, University of Warmia and Mazury in Olsztyn, 10-957 Olsztyn, Poland; 2 Department of Human Physiology and Pathophysiology, School of Medicine, University of Warmia and Mazury in Olsztyn, 10-719 Olsztyn, Poland; 3 Department of Nephrology and Hypertension, University Hospital Erlangen, Friedrich Alexander University Erlangen Nuremberg (FAU), 91054 Erlangen, Germany

**Keywords:** vitamin K, glucose metabolism, type 2 diabetes, osteocalcin, calcium metabolism, endocrine system

## Abstract

Vitamin K (VK), traditionally recognized for its role in coagulation, is increasingly implicated in extrahepatic processes, including glucose metabolism and calcium regulation. A suboptimal VK status is common in the general population and may limit these functions, yet evidence linking VK to glucose metabolism and other endocrine axes remains heterogeneous and incompletely synthesized. This narrative review integrates mechanistic, observational, and interventional evidence to examine the role of VK across the endocrine system, with particular emphasis on glucose metabolism. Mechanistic studies indicate that VK supports pancreatic β-cell function, modulates peripheral insulin sensitivity, and facilitates proper calcium distribution. Observational studies consistently associate a higher VK status with a lower risk of type 2 diabetes, while interventional studies suggest that VK supplementation may improve glucose metabolism, primarily in metabolically impaired populations. In bone and mineral metabolism, VK acts synergistically with calcitriol, with combined supplementation showing more consistent benefits in skeletal outcomes than either vitamin alone. Evidence for VK involvement in other endocrine axes, including reproductive and inflammatory pathways, remains limited and largely mechanistic. Overall, the available evidence supports a context-dependent role for VK in glucose metabolism, influenced by baseline nutritional and metabolic status and outcome selection, as well as a synergistic interaction with calcitriol and parathormone in calcium regulation. Future clinical studies should incorporate baseline VK status stratification, dynamic measures of insulin sensitivity, and adequately powered designs to clarify the therapeutic relevance of VK across endocrine and metabolic outcomes.

## 1. Introduction

Vitamin K (VK) is best known for its essential role in blood coagulation but is increasingly recognized as a contributor to a range of extrahepatic processes, including glucose regulation and insulin sensitivity [1,2], bone and mineral metabolism [3,4], immune and inflammatory pathways [5,6], and vascular health [7,8]. While minimal intake is sufficient to maintain normal coagulation, many of these extrahepatic functions appear to require a higher VK status [9,10,11]. Nevertheless, current dietary reference intakes and clinical practice remain focused on coagulation requirements [9,12], with supplementation recommendations largely limited to neonatal prophylaxis [13,14] and the use of menaquinone-4 in osteoporosis management in Japan [15]. Consequently, a substantial proportion of the population is thought to have a subclinical VK deficiency—a state in which coagulation is preserved but VK availability may be insufficient to support its broader physiological roles [9,10,11,12,16]. Accumulating evidence links this condition to impaired glucose [17,18,19,20,21] and calcium metabolism [4,7], highlighting the need to better define VK’s roles within the endocrine regulatory pathways.

Several reviews have addressed VK and glucose metabolism; however, the most recent synthesis integrating mechanistic insights with human observational and interventional data was published in 2021 [2] and does not incorporate recent advances. Since then, new mechanistic insights have emerged regarding VK-dependent regulation of β-cell function [22,23], peripheral glucose metabolism [24], and antioxidant pathways [25], alongside a substantial increase in interventional studies published in the past five years [26,27,28,29,30,31]. In parallel, reported clinical findings remain heterogeneous, and prior reviews have largely summarized outcomes without systematically addressing key biological and methodological factors—such as baseline VK status, metabolic phenotype, VK form, and outcome selection—that may underlie inconsistent results across studies. Furthermore, the literature on VK and endocrine function remains fragmented, with most reviews focusing on individual domains—such as glucose metabolism, calcium homeostasis, or the proposed endocrine role of osteocalcin—rather than considering endocrine regulation as an integrated system [2,3,32,33,34].

In this narrative review, we integrate the current evidence on VK within the context of the endocrine system, with primary emphasis on glucose metabolism. We synthesize recent mechanistic findings with observational and interventional human data to examine how VK may influence pancreatic β-cell function, insulin secretion, and peripheral insulin sensitivity. We also provide a concise overview of the established and emerging roles of VK in other endocrine pathways and propose a framework for interpreting heterogeneous findings in light of key biological and methodological considerations.

## 2. Materials and Methods

This narrative review examines the endocrine and metabolic roles of VK. The review is based on original research articles, systematic reviews, and meta-analyses published in English. A literature search was conducted using PubMed and Google Scholar from the database’s inception through January 2026. Initial broad searches were conducted to identify endocrine pathways potentially linked to VK, and search terms were subsequently refined based on the availability and relevance of mechanistic and clinical evidence. These included Medical Subject Headings and free-text keywords, such as “vitamin K”, “VK”, “phylloquinone”, “menaquinone”, “osteocalcin”, “OCN”, “endocrin*”, “pancrea*”, “insulin”, “glucose”, “diabetes”, “type 2 diabetes”, “calcitriol”, “vitamin D”, “bone”, “osteoporosis”, “calcium”, “inflammat*”, “metaboli*”, “thyroid”, “parathyroid”, “gonad*”, “testi*”, “ovar*”, “PTH”, “testosterone”, “estrogen”, and “progesterone”, used alone or in combination. The reference lists of relevant articles were additionally screened to identify further studies.

The keywords listed above are illustrative rather than exhaustive and reflect the domains ultimately covered in the manuscript. Additional hormonal axes, including glucagon, cortisol, adipokines, myokines, ADH, oxytocin, adrenal hormones, and melatonin, were considered during the initial search but were not included in the final synthesis due to limited evidence linking these pathways to VK-dependent mechanisms.

We focused on studies examining VK status, intake, or supplementation in relation to endocrine or metabolic outcomes. Human studies were prioritized where available, particularly randomized controlled trials (RCTs) and meta-analyses, while animal and in vitro studies were included to describe mechanistic pathways and support biological plausibility. Evidence was synthesized qualitatively, and no formal risk-of-bias assessment or quantitative synthesis was performed. Where findings were inconsistent, studies were interpreted in the context of study design, population characteristics, and consistency across different lines of evidence. Studies primarily addressing coagulation, cardiovascular endpoints, advanced chronic kidney disease, cancer biology, or pharmacokinetics were not considered unless they directly informed endocrine or metabolic mechanisms.

## 3. Vitamin K Forms and Cellular Mechanisms of Action

### 3.1. VK Vitamers: Sources, Metabolism, and Tissue Availability

VK comprises a group of fat-soluble vitamers sharing a 2-methyl-1,4-naphthoquinone (menadione) core, and is present in the diet as phylloquinone (vitamin K1, VK1) and menaquinones (VK2; MK-n, where n denotes the number of prenyl units in the side chain) [35,36]. VK1 is abundant in leafy green vegetables, whereas MKs are derived mainly from fermented foods and animal products, with MK-4 additionally synthesized endogenously from VK1 [36,37,38,39]. 

Although VK1 accounts for the majority of dietary intake [35,36], it is tightly bound to plant tissue and is less efficiently absorbed than MKs [35,37,40]. Following intestinal absorption, VK1 is transported predominantly in triacylglycerol-rich lipoproteins and is taken up by the liver, resulting in a short biological half-life [36,39,41]. In contrast, MKs preferentially associate with low-density lipoproteins, which limits hepatic uptake and confers longer circulation times and greater availability to extrahepatic tissues [35,36,39,41]. Consistent with these pharmacokinetic differences, MKs surpass VK1 levels in most peripheral tissues, and likely contribute disproportionately to VK’s extrahepatic functions [37,39,41,42]. 

In addition to dietary forms, VK also includes hydrophilic precursors such as menadione (vitamin K3) and its derivatives (vitamin K4) [35]. Menadione serves as an intermediate in the conversion of VK1 to MK-4, while VK4 refers to synthetic menadione precursors [27,35,41]. Although these compounds can substitute for VK, their use in humans is limited by safety concerns at higher concentrations [11,35,43,44].

### 3.2. Cellular Actions of VK: γ-Carboxylation, Signaling, and Radical Scavenging

At the cellular level, VK acts through three principal mechanisms. First, it functions as a coenzyme for γ-glutamyl carboxylase (GGCX), enabling the post-translational conversion of glutamate (Glu) residues into γ-carboxylglutamyl (Gla) residues [35,39]. Approximately 20 proteins undergo this modification and are collectively termed VK-dependent proteins (VKDPs) [22,45,46,47,48,49,50,51,52,53,54,55,56,57,58,59,60]. The resulting Gla residues confer high-affinity calcium binding, which is essential for VKDP function.

Second, VK modulates several intracellular signaling pathways, including AMP-activated protein kinase (AMPK) [19,24], the cAMP-dependent exchange protein directly activated by cAMP 2 (Epac2), and protein kinase A (PKA) [61,62,63,64,65]. These pathways are directly relevant to endocrine function and are discussed in subsequent sections. VK has also been shown to influence several transcription factors [4,66,67,68,69,70] and cell-survival pathways [11,67,71,72,73]; however, the endocrine relevance of these actions remains unknown.

**Figure 1:**
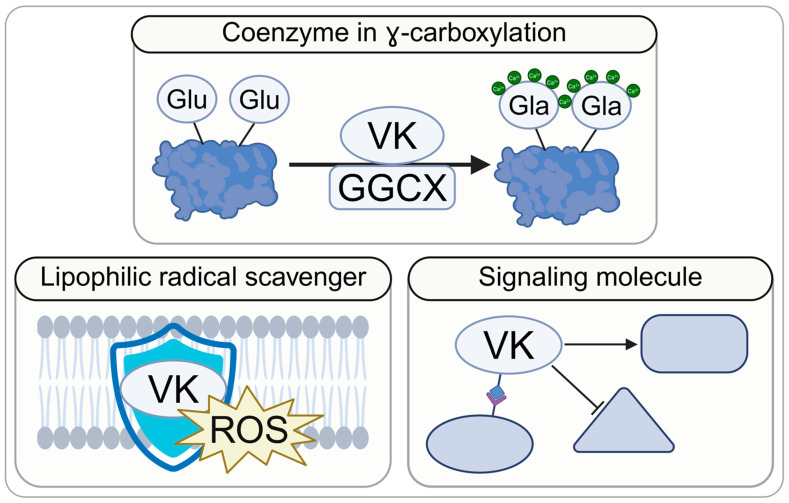
The three modes of vitamin K action. Notes: A more detailed mechanistic overview is provided in Supplementary Figure S1. Created in BioRender. Madeksza, M. (2026) https://BioRender.com/cjey5vz. Abbreviations: GGCX, γ-glutamyl carboxylase; Gla, γ-carboxylglutamyl acid; Glu, glutamate; ROS, reactive oxygen species; VK, vitamin K.

Finally, VK acts as a lipophilic radical-trapping antioxidant that inhibits (phospho)lipid peroxidation and suppresses ferroptosis, an iron-dependent form of cell death driven by lipid peroxidation [25,74,75]. Figure 1 provides a simplified overview of these mechanisms, while a more detailed representation is presented in Supplementary Figure S1.

## 4. Vitamin K Status and Its Measurement

### 4.1. VK Status Markers, Their Use, and Limitations

The assessment of VK nutritional status relies on two primary approaches: dietary questionnaires and biomarkers [76]. Dietary questionnaires are practical and cost-effective, making them common in large observational studies [77], but they are subject to recall bias and confounding by overall diet quality and lifestyle, as phylloquinone intake often reflects the consumption of leafy greens and other health-associated foods [78,79,80]. Although adjustment for dietary and lifestyle factors can mitigate this bias, it cannot fully eliminate it [78].

Biomarkers provide a more objective assessment of VK status and include circulating VK concentrations, functional measures of VKDP carboxylation, coagulation assays, and urinary metabolites [78]. Circulating VK1 reflects recent intake, peaking approximately 6–10 h postprandially, and is influenced by triglyceride levels due to transport on triacylglycerol-rich lipoproteins; accordingly, fasting samples and adjustment for triglycerides are recommended [78]. 

Undercarboxylated osteocalcin (ucOCN) and dephosphorylated-undercarboxylated matrix Gla protein (dp-ucMGP) are the most sensitive indicators of extrahepatic VK status [78,81]. Elevated levels of these proteins reflect low VK availability in tissues where they are predominantly active—primarily bone for ucOCN and vascular smooth muscle for dp-ucMGP. Expressing these measures relative to total protein concentrations (e.g., %ucOCN) improves interpretability, although they are not entirely VK-specific: their levels are influenced by physiological factors such as bone turnover (ucOCN) [78,82,83,84] and age or vascular pathology (dp-ucMGP) [78,85]. Both %ucOCN and dp-ucMGP respond rapidly to changes in VK intake and therefore primarily reflect short-term VK status [82,86,87,88].

Other VKDP-based measures, including undercarboxylated prothrombin and coagulation assays, are less sensitive within physiological VK ranges, whereas urinary VK metabolites are impractical for large-scale studies [78]. Overall, no single gold-standard biomarker is currently available, and a combined assessment of circulating VK1 and a functional VKDP marker (%ucOCN or dp-ucMGP) offers the most comprehensive evaluation of VK status [78]. Supplementary Table S1 summarizes the available VK status markers and their key limitations.

### 4.2. Genetic Determinants of VK Status

VK status markers show large interindividual variability compared with other fat-soluble vitamins. This variability is only partly explained by dietary intake and other nongenetic factors, and several single nucleotide polymorphisms (SNPs) have been associated with VK status (Table 1) [20,89,90]. These variants cluster in genes involved in VK metabolism (e.g., *CYP4F2*, *VKOR*, and *GGCX*) [91,92], lipoprotein-mediated transport (e.g., the *APOA1/C3/A4/A5* gene cluster) [41,90], as well as additional loci whose functional roles in VK biology remain unclear [89]. 

Consistent with a genetic contribution, a multi-ethnic cohort has identified race and ethnicity as significant predictors of circulating VK1 concentrations, with higher levels observed in Chinese American and African American populations compared with Caucasian and Hispanic populations [93]. These findings suggest that genetic background substantially influences VK status and responsiveness to VK intake, which may predispose certain individuals to low functional VK availability despite similar dietary exposure [86,89,90].

**Table 1:** Genetic polymorphisms associated with VK status.

Gene(s)	Encoded Protein(s)	SNP(s)	Effect on VK Status	Ref.
*CYP4F2*	CYP4F2 (VK1 oxidase)	rs2108622	↑ plasma VK1 and MK-4	[94]
*VKORC1*	VKOR	rs8050894	↑ plasma VK1	[95]
*GGCX*	GGCX	rs10187424, rs7568458	↑ %ucOCN	[95]
*APOA1/C3/A4/A5* cluster	apolipoproteins	rs964184	↑ plasma VK1	[90]
*CTNNA2*	α-2-catenin	rs2192574, rs4852146	↑ plasma VK1	[90]
*CDO1*	cysteine dioxidase 1	rs4122275, rs686207, rs6862909	↓ plasma VK1	[90]
*COL22A1*	collagen	rs2199565, rs4645543, rs7018214	↓ plasma VK1	[90]

Note: ↑ indicates an increase and ↓ indicates a decrease in the specified VK status biomarker associated with the given genetic polymorphism(s). Abbreviations: GGCX, γ-glutamyl carboxylase; SNP, single nucleotide polymorphism; VK1, vitamin K1 (phylloquinone); VKOR, vitamin K epoxide reductase; %ucOCN, % of undercarboxylated osteocalcin.

### 4.3. Baseline VK Status and Responsiveness to Supplementation

As with many micronutrients [96,97,98], the biochemical and clinical effects of VK supplementation appear to be greatest in individuals with a low baseline status, while those with adequate VK levels show limited responses due to a ceiling effect [5,99,100,101,102,103,104,105]. Consequently, supplementation trials that do not account for baseline VK status may fail to detect meaningful benefits confined to deficient subgroups. These and other factors complicating the interpretation of clinical VK research are further discussed in Section 8.4.

## 5. Vitamin K and Glucose Metabolism: Mechanistic Plausibility and Clinical Evidence from Human Studies

### 5.1. VK and β-Cell Function: ER Calcium Handling, cAMP Signaling, and Inflammatory Stress

VK supports β-cell integrity and function through ER calcium handling, cAMP signaling, and anti-inflammatory effects. Endoplasmic reticulum Gla protein (ERGP) is a recently identified VKDP that restrains store-operated calcium entry (SOCE) and protects β-cells from dysregulated insulin secretion under metabolic stress [22]. The tight regulation of intracellular Ca^2+^ is essential for β-cell function, as Ca^2+^ influx triggers insulin granule exocytosis [106,107,108]. SOCE replenishes intracellular Ca^2+^ following the depletion of endoplasmic reticulum (ER) Ca^2+^ stores and supports β-cell integrity and glucose-stimulated insulin secretion (GSIS) [109,110]. However, chronic nutrient excess induces ER stress and ER Ca^2+^ depletion [108,111], which can lead to excessive SOCE activation [112], uncontrolled insulin secretion, and downstream insulin resistance [22,113,114]. ERGP counteracts this process in a glucose-dependent manner: rising intracellular glucose levels enhance ERGP γ-carboxylation, strengthening its inhibitory effect on SOCE and preventing Ca^2+^ overflow [22]. The physiological relevance of this mechanism is underscored by β-cell-specific *Ggcx*-knockout mice, which exhibit β-cell apoptosis and impaired GSIS, and develop insulin resistance with fasting hyperinsulinemia and hyperglycemia within one week of a high-fat diet [22]. These findings suggest that VK-dependent regulation of ER Ca^2+^ handling helps preserve β-cell function under conditions of metabolic stress.

Beyond ER calcium homeostasis, VK can also influence β-cell function through cAMP signaling. In isolated islets and INS-1 cells, MK-4 enhances GSIS via increased intracellular cAMP levels and the activation of the exchange protein directly activated by the cAMP 2 (Epac2) pathway [23]. Because the cAMP/Epac2 axis contributes to the incretin-dependent potentiation of GSIS without increasing basal insulin secretion [115,116,117,118], activation of this pathway by VK may help preserve incretin responsiveness, which is impaired in type 2 diabetes (T2D) [119,120].

In addition to these signaling mechanisms, VK participates in multiple anti-inflammatory and antioxidant pathways across diverse cell types, as detailed in the Supplementary Figure S1 [25,36,66,74,75,91,121,122,123,124,125,126,127,128,129,130,131,132,133,134,135,136,137,138,139]. In the pancreas, VK1 administration in a streptozotocin-induced diabetes model reduced oxidative stress, preserved islet morphology, and restored insulin secretion [140]. Although this model reflects toxin-induced β-cell injury rather than the pathophysiology of T2D, it provides direct evidence that VK can modulate inflammatory signaling within the endocrine pancreas. Collectively, these mechanisms support the plausibility that an adequate VK status contributes to β-cell integrity and adaptive insulin secretion during metabolic stress, consistent with the high concentrations of vitamin K observed in pancreatic tissue.

### 5.2. VK and Insulin Resistance: Direct and Indirect Links

Beyond its effects on β-cell function, VK has been shown to alleviate hepatic and peripheral insulin resistance through tissue-intrinsic metabolic mechanisms. In mouse models of T2D, VK1 supplementation improves impaired glucose metabolism through the activation of sirtuin 1 (SIRT1)-dependent pathways in the liver [19], while VK2 supplementation enhances insulin sensitivity, mitochondrial function, and oxidative fiber composition in skeletal muscle via the same signaling axis [24]. Notably, VK1—the predominant dietary form of VK—is preferentially taken up by the liver [9,42]; thus, hepatic VK actions (e.g., SIRT1 pathway activation) are supported across a broad range of intakes, whereas VK-mediated mechanisms in peripheral tissues such as skeletal muscle may require higher intake and are therefore more sensitive to variations in VK status.

**Figure 2:**
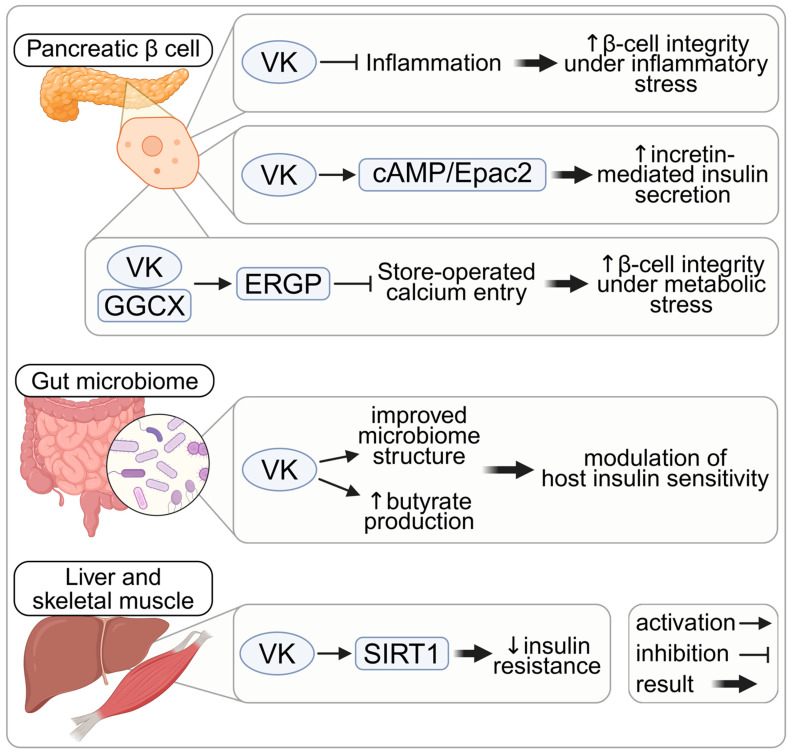
Mechanisms by which vitamin K may influence glucose metabolism and insulin sensitivity. Notes: ↑ indicates an increase and ↓ indicates a decrease in the indicated biological effect associated with VK action. Created in BioRender. Madeksza, M. (2026) https://BioRender.com/kucipw1. Abbreviations: Epac2, cAMP-dependent exchange protein directly activated by cAMP 2; ERGP, endoplasmic reticulum Gla protein; GGCX, γ-glutamyl carboxylase; SIRT1, sirtuin 1; VK, vitamin K.

Furthermore, VK may indirectly influence glucose metabolism through modulation of the gut microbiome. Several gut commensal bacteria rely on VK as an electron carrier [141], and VK deficiency or warfarin exposure has been associated with microbial dysbiosis in rodent models [142,143]. Conversely, higher dietary VK1 intake has been linked to increased microbial production of butyrate [144]—a short-chain fatty acid associated with improved insulin sensitivity and metabolic health [145,146]. Given that butyrate production is reduced in T2D [147,148,149,150], microbiome-mediated effects may contribute to VK-associated metabolic improvements. Additionally, VK’s involvement in multiple anti-inflammatory and antioxidant pathways (Supplementary Figure S1) may support insulin signaling in peripheral tissues, providing additional biological plausibility for VK-mediated improvements in insulin sensitivity. However, direct evidence for VK-dependent anti-inflammatory effects in insulin-responsive tissues is limited. These β-cell-intrinsic and peripheral actions of VK are summarized in Figure 2.

### 5.3. Osteocalcin and Glucose Metabolism: Mediator, Marker, or Confounder?

OCN is unique among other VKDPs because its undercarboxylated form circulates in blood, where it was proposed to act as a hormone; however, the nature and relevance of its endocrine role in humans remains incompletely defined [151,152]. Observational studies consistently associate higher circulating OCN levels with favorable metabolic outcomes, including a lower prevalence of T2D [153,154], obesity [154,155], and metabolic syndrome [153,154], as well as lower HbA1c, fasting and OGTT blood glucose [153,154], reduced HOMA-IR [153], improved β-cell function [154], and higher adiponectin levels [154]. Importantly, fewer than one-third of these studies distinguish between different OCN forms [82,154,155]. Among those that do, both total OCN and ucOCN generally correlate with metabolic outcomes [154,155,156], and a contribution from the carboxylated form cannot be excluded [157,158,159,160,161,162,163,164]. Moreover, circulating OCN levels are strongly influenced by bone remodeling activity [82,165], which itself is altered in metabolic disease [166,167], raising the possibility that OCN acts primarily as a biomarker or confounder rather than a direct endocrine mediator. Preclinical studies have not resolved this uncertainty [81].

In this context, the interpretation of VK’s effects is further complicated by the fact that VK supplementation typically reduces %ucOCN without altering total OCN [81,168,169]. As long as the metabolically active OCN form(s) in humans remain undefined, it is therefore unclear whether changes in OCN carboxylation represent a mechanistic pathway linking VK to glucose metabolism or an epiphenomenon reflecting altered bone turnover [82].

### 5.4. VK Status and Risk of T2D: Observational Evidence

In line with the preclinical findings described above, a growing body of clinical research suggests an association between VK status and glucose metabolism, particularly under conditions of metabolic impairment. Three large observational studies, including one Mendelian randomization analysis, reported inverse associations between VK status and risk of T2D [18,20,170], while two additional studies found that warfarin use—unlike anticoagulants not targeting γ-carboxylation—was associated with a higher risk of T2D [171,172]. These results suggest the potential protective role of an adequate VK status in the development of T2D.

### 5.5. VK and Glucose Metabolism: Effects Depend on Baseline Metabolic Status and Outcome Measures

Evidence from intervention and observational studies indicates that the effects of VK on glucose metabolism are heterogeneous and dependent on baseline metabolic status (Table 2). Across 20 identified studies (five observational, 15 interventional), benefits were consistently observed in individuals with impaired glucose metabolism but not in metabolically healthy populations. In line with a 2018 meta-analysis reporting no effect of VK supplementation in healthy individuals [173], studies conducted in normoglycemic participants generally failed to demonstrate improvements in glycemic control [16,174,175,176]. In contrast, all nine studies involving participants with (pre)diabetes or polycystic ovary syndrome (PCOS) reported significant improvements in two or more glucose metabolism parameters with a higher VK status or supplementation.

The apparent efficacy of VK also varies by outcome measure. Fasting plasma glucose was largely unaffected, while effects on fasting insulin and HOMA-IR were inconsistent, with roughly equal numbers of studies reporting reductions or null findings. This pattern is reflected in meta-analytic evidence: a 2017 meta-analysis limited to non-diabetic individuals found no significant effects of VK supplementation on fasting insulin or HOMA-IR [177], whereas two 2025 meta-analyses that included participants with (pre)diabetes and PCOS reported significant reductions in fasting insulin, HOMA-IR, and HbA1c [1,21]. 

More consistent improvements were observed for dynamic indices of insulin sensitivity: ISI(0,120) improved in all four studies assessing it [178,179,180,181], QUICKI improved in four of five studies [17,29,30,182,183], and OGTT plasma glucose improved in three of four studies [27,178,181,184]. The observation that ISI(0,120)—capturing both hepatic and peripheral glucose disposal—showed more consistent improvement than HOMA-IR, which primarily reflects hepatic insulin resistance, suggests that VK may preferentially enhance peripheral insulin sensitivity [181,185,186]. However, the limited number of studies and substantial heterogeneity in study design preclude definitive conclusions.

**Table 2:** A summary of human studies on the effect of VK on insulin sensitivity and/or glycemic status.

Study	Year	Subjects (N)	VK Dose/VK Status	Period	Outcome
(a) observational studies					
Santos et al. [17]	2020	Adult men (136) and women (192)	Usual dietary intake	Cross-sectional study	in women: (Higher VK intake) ↓ fasting PG, ↓ fasting PI, ↓ HOMA-IR, ↑ QUICKI; ↔ in men
Zwakenberg et al. [20]	2019	Pooled from: the EPIC cohort study, the DIAGRAM meta-analysis, and the UK Biobank cohort (total of 69,647 adults with T2D)	Usual dietary intake	EPIC cohort: 1997–2007; UK Biobank cohort: 2006–2010; DIAGRAM meta-analysis: data from 23 studies	(Higher genetically predicted circulating VK1) ↓ T2D risk
Dihingia et al. [19]	2018	Adult men and women with T2D (25) and healthy controls (20)	Usual dietary intake	Cross-sectional study	↓ circulating VK1 in T2D vs. controls (Higher circulating VK1) ↓ fasting PG, ↓ HOMA-IR among T2D patients
Ibarrola-Jurado et al. [18]	2012	Elderly men (861) and women (1062) with high CV risk	Usual dietary intake	5.5 years (median follow-up)	(Higher VK1 intake) ↓ T2D risk
Beulens et al. [170]	2010	Adult men (9740) and women (28,354)	Usual dietary intake	10.3 years	(Higher VK intake) ↓ T2D risk
Pan and Jackson [16]	2009	Adult men (2867) and women (2933)	Usual dietary intake	Cross-sectional study	(Higher VK intake) ↔ fasting PG and fasting PI
Yoshida et al. [178]	2008	Adult men (1247) and women (1472)	Usual dietary intake	1 year	(Higher VK intake) ↓ OGTT PG, ↑ ISI(0,120) ↔ fasting PG, HbA1c, PI, and HOMA-IR
Sakamoto et al. [184]	1999	Healthy young men (16)	Usual dietary intake	1 week food checklist, acute insulin response	(Higher VK intake) ↓ OGTT PG, ↑ insulin response ↔ fasting PG
(b) interventional studies					
Zhang et al. [26]	2023	Adult men (29) and women (31) with T2D	With or without 90 μg/day MK-7	6 months	↓ fasting PG, ↓ fasting PI, ↓ HbA1c, ↓HOMA-IR
Ali et al. [27]	2023	Adult men (10) and women (80) with T2D	With or without 1000 μg/day K4	6 months	↓ fasting PI, ↓ HOMA-IR; ↓ treatment intensification, ↑ dose reduction of antidiabetic drugs; ↔ fasting PG and OGTT PG
Adeli et al. [28]	2023	Adult men and women with T2D (45)	With or without 200 μg/day MK-7	3 months	↓ fasting PG, ↓ fasting PI; ↓ plasma leptin
Rahimi Sakak et al. [29]	2021	Adult men and women with T2D (63)	With or without 360 μg/day MK-7	3 months	↓ fasting PG, ↓ HbA1c; ↔ fasting PI, HOMA-IR, QUICKI
Karamzad et al. [31]	2020	Adult men (31) and premenopausal women (14) with T2D	With or without 200 μg/day MK-7	3 months	↓ fasting PG and HbA1c; ↔ fasting PI, HOMA-IR
Tarkesh et al. [30]	2020	Women with PCOS (79)	With or without 90 μg/day MK-7	2 months	↓ HOMA-IR, ↓ PI, ↑ QUICKI ↔ fasting PG
Karamali et al. [182]	2017	Women with PCOS (55)	With or without 180 μg MK-7, 10 μg vitamin D and 1 g Ca/day	2 months	↓ PI, ↓ HOMA-IR, ↑ QUICKI ↔ fasting PG
Asemi et al. [183]	2016	Adult men (35) and women (31) with T2D and coronary heart disease	With or without 180 μg MK-7, 10 μg vitamin D and 1 g Ca/day	3 months	↓ PI, ↓ HOMA-IR, ↑ QUICKI ↔ fasting PG
Knapen et al. [176]	2015	Healthy postmenopausal women (244)	With or without 180 μg/day MK-7	3 years	↔ fasting PG
Centi et al. [174]	2015	Healthy adult men and women (42)	500 μg/day VK1	1 month	↔ on HOMA-IR
Rasekhi et al. [181]	2015	Premenopausal prediabetic women (82)	With or without 1000 μg/day VK1	1 month	↓ OGTT PG, ↓ OGTT plasma insulin, ↑ ISI(0,120); ↔ on fasting PG, fasting PI, and HOMA-IR
Choi et al. [179]	2011	Healthy young men (33)	With or without 30,000 µg/day MK-4	1 month	↑ ISI(0,120); ↔ on fasting PG
Kumar et al. [175]	2010	Healthy postmenopausal women (42)	With or without 1000 μg/day VK1	1 year	↔ fasting PG, fasting PI, and HOMA-IR
Yoshida et al. [178]	2008	Elderly nondiabetic men (124) and women (165)	With or without 500 μg/day VK1	3 years	↓ fasting PI and ↓ HOMA-IR in men; ↔ in women; ↔ fasting PG in both sexes
Sakamoto et al. [180]	2000	Healthy young men with low (4) medium (4) and high (4) DP levels	90,000 µg/day MK-4	1 week	↑ ISI(0,120) in men with high DP levels; ↔ on ISI in other subjects; ↔ on all other measured parameters

↓—VK supplementation (or higher serum concentration) resulted in a significant decrease in a parameter; ↑—it resulted in a significant increase in a parameter; ↔—it had no significant effect on a parameter. Abbreviations: CV, cardiovascular; DP, decarboxylated prothrombin; DIAGRAM, Diabetes Genetics Replication and Meta-analysis; EPIC, European Prospective Investigation into Cancer and Nutrition; OGTT, oral glucose tolerance test; PG, plasma glucose; PI, plasma insulin; ISI, insulin sensitivity index; HOMA-IR, homeostatic model assessment for insulin resistance; T2D, type 2 diabetes; PCOS, polycystic ovary syndrome.

### 5.6. Other Cardiometabolic Domains Linked to VK Status

Beyond glycemic control, VK status has been associated with several cardiometabolic domains relevant to T2D, although evidence in these areas is largely observational or derived from preclinical models. Cross-sectional studies consistently report inverse associations between VK status and measures of adiposity [163,187,188,189], circulating inflammatory markers [5,16,170,190,191,192,193,194], and a more favorable lipid profile [16,170,189,195]. Experimental and cellular studies also suggest vasoprotective effects, including improved NO-dependent endothelial function [196,197,198], anti-senescence effects on endothelial and vascular smooth muscle cells, and the inhibition of glucose-induced endothelial inflammation [121,198]. However, RCTs have generally failed to demonstrate corresponding effects of VK supplementation [21,27,29,31,177,199,200]. Collectively, these findings suggest that VK may influence multiple cardiometabolic pathways relevant to T2D, but current evidence does not support a clear causal effect of VK supplementation on these outcomes. The implications and limitations of this evidence are considered in greater detail in Section 8.3.

## 6. Vitamin K Collaborates with PTH and Calcitriol to Ensure Proper Calcium Distribution

### 6.1. VK Facilitates Safe Calcium Mobilization by Parathormone and Calcitriol

Parathormone (PTH) and calcitriol are the principal regulators of systemic calcium balance, acting on bone, the kidney, and the intestine to increase circulating calcium levels [201,202,203,204]. Both hormones also induce the expression of OCN and MGP [122,205,206,207,208,209,210], creating a functional dependence on adequate VK availability.

VK-dependent γ-carboxylation converts OCN and MGP into their biologically active forms, thereby directing the fate of mobilized calcium. Carboxylated OCN binds hydroxyapatite crystals in bone, regulating their shape, size, and alignment with collagen to support optimal bone strength and architecture [3,211,212], whereas phosphorylated-carboxylated MGP inhibits calcium-phosphate crystal growth in soft tissues and the vasculature [122,123,124]. Thus, by increasing the proportion of active OCN and MGP, a sufficient VK status ensures that calcium mobilized by PTH and calcitriol is deposited in bone rather than ectopically.

### 6.2. VK Amplifies Skeletal Responses to Calcitriol and Parathormone

VK influences bone remodeling by promoting osteoblast activity, suppressing osteoclastogenesis, and reducing %ucOCN [3,168,213,214]. However, RCTs of VK supplementation alone have reported inconsistent effects on bone mineral density (BMD) or fracture risk [169,215,216]. Similarly, calcitriol modestly improves BMD, while its effects on fracture prevention are limited [217,218,219,220].

In contrast, multiple trials indicate that combined VK and calcitriol supplementation produces greater improvements in BMD and fracture outcomes than either vitamin alone [221,222]. Comparable synergistic effects have also been observed when VK is administered alongside recombinant parathormone [223,224,225]. These findings suggest that VK enhances the skeletal efficacy of calcium-regulating hormones, likely by increasing cOCN levels within bone.

## 7. Vitamin K and Other Endocrine Axes

### 7.1. Reproductive Axes: Evidence for Testosterone Synthesis and Limited Data on Estrogen

Beyond glucose and calcium metabolism, experimental evidence also supports a role for VK in androgen synthesis. In Leydig cells, MK-4 enhances testosterone production via activation of the cAMP/PKA pathway, which stimulates steroidogenic acute regulatory protein (StAR)-mediated cholesterol transport and upregulates steroidogenic enzyme expression via transcription factors such as CREB and SF-1 [226,227]. Consistent with this mechanism, VK supplementation increases plasma and intratesticular testosterone levels in rats [63,228,229], and attenuates inflammation-induced suppression of steroidogenesis [230]. Human data remain sparse and inconsistent; few studies have assessed VK status alongside testosterone, reporting both inverse [231] and positive [232] associations with %ucOCN. A related observational study also linked higher VK intake to greater muscle mass in men but not women [233].

Furthermore, total OCN levels correlate positively with testosterone across populations [234,235,236,237,238], and OCN has been proposed as a regulator of male gonadal function [239,240]. However, similarly to what was noted in Section 5.3, few studies distinguished between OCN forms, limiting the interpretation of VK’s role in this potential regulatory axis.

Some evidence also suggests that VK may be involved in female reproductive endocrinology. Ovaries contain high VK concentrations and express VK-cycling enzymes [42,241], and PKA-dependent steroidogenic pathways—upregulated by VK in other tissues [61,62,63,64,209]—are central to ovarian hormone synthesis [242,243,244]. Observationally, lower circulating ucOCN has been associated with higher estrogen levels in women [245,246,247,248], though this relationship may reflect estrogen-mediated suppression of bone turnover rather than direct VK action. Additionally, in vitro, MK-4 upregulates 17β-hydroxysteroid dehydrogenase type 4, favoring estradiol-to-estrone conversion and reduced estrogen receptor activation [249], but the relevance of this mechanism remains unclear. In all, VK may contribute to gonadal steroidogenesis, particularly testosterone synthesis in men, but the evidence remains preliminary.

### 7.2. Thyroid Hormones and Other Endocrine Axes: Indirect and Uncertain Interactions with VK

VK is not known to directly influence the synthesis, metabolism, or action of most hormones. Although OCN has been implicated in the regulation of other endocrine axes, including GLP-1 and neuroendocrine pathways [50,250], the extent to which VK-dependent γ-carboxylation modulates these actions is unclear.

Separately, thyroid axis hormones have been shown to influence the activity of VK-dependent coagulation pathways. In rats, the administration of TRH, TSH, T3, and T4 reduces the activity of VK-dependent clotting factors II, VII, IX, and X [251,252]. This interaction has clinical implications, as thyroid status alters sensitivity to VK antagonists; hyperthyroidism or the initiation of L-thyroxine therapy generally necessitates warfarin dose reduction, whereas hypothyroidism or antithyroid therapy typically requires dose escalation [253,254,255,256].

## 8. Discussion

### 8.1. Key Findings

At the cellular level, VK acts as a coenzyme for γ-carboxylation of VKDPs, a signaling molecule, and a free-radical scavenger. Mechanistic studies demonstrate the VK-dependent regulation of pancreatic β-cell function, as well as effects on insulin sensitivity in peripheral tissues. In observational studies, a higher VK status is consistently associated with a lower risk of T2D, while interventional trials report heterogeneous effects of VK supplementation on glucose metabolism, with benefits observed primarily in metabolically impaired populations. In calcium and bone metabolism, VK interacts with PTH and calcitriol through γ-carboxylation of OCN and MGP, with combined supplementation studies reporting more consistent effects on skeletal outcomes than either intervention alone. Experimental data also suggest VK-dependent modulation of testicular steroidogenesis and inflammatory pathways, but human evidence in these areas is sparse and inconsistent.

### 8.2. Unresolved Mechanistic Questions

Several mechanistic uncertainties remain. The most prominent concerns the proposed endocrine role of OCN. Early mouse models suggested that OCN deficiency leads to hyperglycemia, reduced insulin production, increased visceral fat, and hypogonadism, supporting its classification as a bone-derived hormone [257]. However, more recent *Ocn* knockout lines failed to reproduce these phenotypes [212,258]. Moreover, while ucOCN is considered the active form in mice [259,260,261,262], VK supplementation—which increases OCN carboxylation—has nonetheless been reported to improve glucose metabolism [19,24,26,263] and male reproductive function [264]. Interpretation is further complicated by the fact that circulating OCN reflects bone remodeling activity [82,165], which is reduced in individuals with diabetes [166,167]. Consequently, associations between OCN and metabolic outcomes may be confounded by altered bone turnover rather than reflecting direct endocrine signaling [82]. To resolve these discrepancies, future clinical studies should distinguish between total and undercarboxylated OCN and incorporate additional markers of bone remodeling. Combined with emerging in vitro and omics-based approaches [151], this will help clarify the endocrine relevance of specific OCN forms in humans. 

Another uncertainty concerns the role of VK’s anti-inflammatory actions in endocrine signaling. While it remains unclear whether VK supplementation can reduce systemic inflammation [177,179,200], its involvement in multiple anti-inflammatory and antioxidant pathways (Supplementary Figure S1) may plausibly improve endocrine signaling by limiting inflammation within endocrine and effector tissues. For example, VK attenuates LPS-induced inflammation and preserves testosterone synthesis in rat testes [230], and in the pancreas, it reduces streptozotocin-induced oxidative stress, preserves islet morphology, and restores insulin secretion [140]. Comparable studies in other endocrine tissues are lacking, representing a notable gap, given that inflammation commonly affects endocrine organs, including the ovaries in PCOS [265,266], and the thyroid gland in autoimmune thyroid disease [267,268].

Finally, while VK’s insulin-sensitizing effects have been established in the liver and skeletal muscle [19,24], its actions within white adipose tissue (WAT) remain largely unexplored [269]. Importantly, WAT is an adipokine-secreting endocrine organ whose dysfunction plays a central role in the metabolic inflammation associated with obesity and insulin resistance [270]. Given VK’s anti-inflammatory properties and its ability to activate AMPK, VK may plausibly improve both insulin sensitivity and adipokine secretion in WAT, representing a priority for future research.

### 8.3. Quality and Interpretation of Clinical Evidence

**Figure 3:**
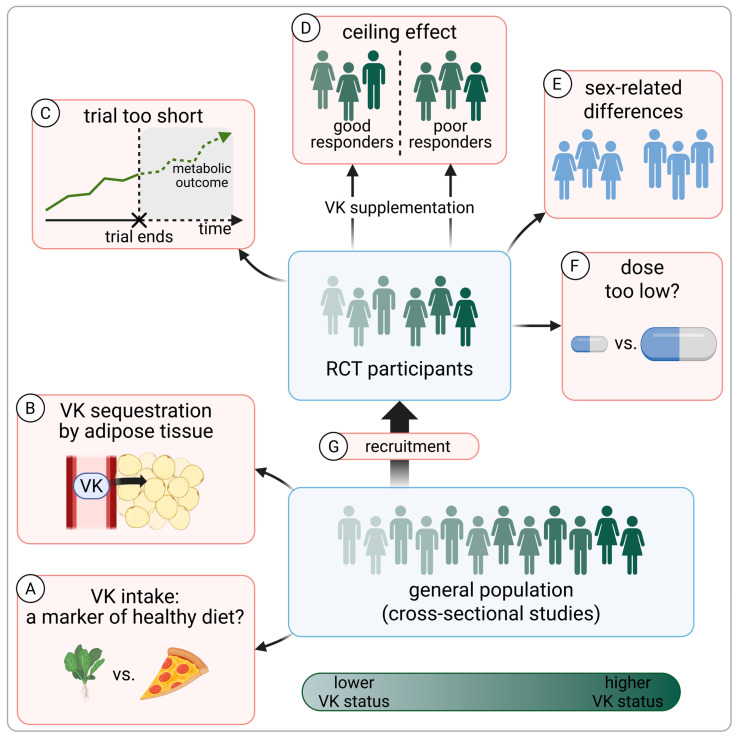
Factors complicating clinical research on vitamin K. Notes: Points (**A**–**F**) are covered in more detail in Section 8.4. (**A**)—VK intake is often assumed to reflect healthier dietary patterns; however, mixed meals and convenience foods are major contributors to VK1 intake due to vegetable oil content, while VK2 is derived primarily from cheese, other dairy products, and meat. (**B**)—VK can be sequestered in adipose tissue, potentially reducing its bioavailability in individuals with a higher body fat percentage. (**C**)—Metabolic changes occur gradually, and many trials may have been too short to detect meaningful effects. (**D**)—The biochemical and clinical effects of VK supplementation depend on baseline VK status; a ceiling effect in individuals with an adequate status may obscure benefits associated with correcting a suboptimal VK status. (**E**)—The available RCT evidence includes an underrepresentation of men, in whom VK may act through distinct mechanisms and for whom supplementation effects may differ from those observed in women. (**F**)—The dose and form of VK used in some trials may have been insufficient to elicit extrahepatic effects. (**G**)—Trial participants are often health-conscious volunteers and therefore less likely to have low nutrient intakes, increasing the proportion of individuals susceptible to ceiling effects [98]. Created in BioRender. Madeksza, M. (2026) https://BioRender.com/xsnia3i. Abbreviations: VK, vitamin K.

While mechanistic studies provide important biological context, the clinical relevance of vitamin K supplementation in humans must ultimately be based on clinical evidence. Within clinical data, a recurring challenge is the discrepancy between cross-sectional findings and RCTs. Observational studies consistently associate a higher VK status with better bone health [271], a lower risk of T2D (Table 2) and cardiovascular disease [272], lower inflammatory markers [5,16,170,190,191,192,193,194], and lower adiposity [163,187,188,189,273], whereas RCTs often fail to demonstrate comparable effects of VK supplementation. Null RCT findings are frequently interpreted as evidence against a causal role of VK, while observational associations are commonly attributed to residual confounding, under the assumption that VK-rich diets reflect healthier lifestyle patterns [2,20,273]. However, this interpretation is overly simplistic and overlooks several additional factors, summarized in Figure 3.

First, population-based dietary surveys challenge the notion that VK intake is a reliable marker of health-conscious behavior. In NHANES 2011–2012, mixed meals and convenience foods were major contributors to phylloquinone intake, presumably due to vegetable oil content [274,275]. Among individuals with low vegetable intake—representing approximately three-quarters of the cohort—VK1 intake from mixed meals, fats, oils, and snacks exceeded that from vegetables. Similarly, in Western diets, menaquinones are derived predominantly from cheese, other dairy products, and meat, which are not typically associated with overall healthier dietary patterns [36,170]. These findings weaken the assumption that VK intake simply proxies diet quality.

On the other hand, VK’s lipophilic nature may help explain some cross-sectional associations without implying causality. Like other fat-soluble vitamins, VK can be sequestered in adipose tissue, potentially reducing its bioavailability in individuals with a higher body fat percentage [187]; this may partly account for inverse associations between VK status markers and adiposity [163,187,188,189], as well as downstream associations with insulin resistance, inflammation, and cardiometabolic risk.

Furthermore, several methodological limitations of existing RCTs likely reduce their ability to detect the benefits of VK supplementation. Many trials may be too short to capture changes in outcomes that evolve gradually, such as body composition, inflammation, or insulin sensitivity. Notably, although most trials assessing anthropometric outcomes reported null findings [15,26,27,28,29,30,103,163,181,276,277,278,279], the few studies with longer follow-up durations (3 years) did demonstrate significant effects [103,163]. 

Another key issue is the baseline VK status. The biochemical and clinical effects of VK supplementation appear to be greatest in individuals with a low baseline status, while those with adequate VK levels show limited responses due to a ceiling effect [5,99,100,101,102,103,104,105]. Despite this, few trials in endocrine and cardiometabolic research have stratified participants by baseline VK status or conducted responder analyses [103,180], raising the possibility that overall null results obscure clinically meaningful effects confined to deficient subgroups.

An additional limitation may be sex imbalance. As shown in Table 2, the available RCT evidence on VK and glucose metabolism is characterized by a predominance of female participants, and similar patterns have been reported across other areas of VK research [21,280]. As discussed earlier, sex-related differences in VK metabolism and actions are likely, and the current interventional data may therefore insufficiently capture VK-related mechanisms that are more relevant in men, including potential effects on testosterone synthesis and muscle metabolism.

Finally, the dose and form of VK used in several trials may have been suboptimal for eliciting extrahepatic effects. Current dietary reference intakes for VK1—90 μg/day for women and 120 μg/day for men—are based solely on coagulation requirements, reflecting VK action in the liver [9]. Because VK1 is preferentially taken up by the liver, substantially higher intakes may be necessary to support extrahepatic tissues [36,39,41], and even the dose of 500 μg/day of VK1 used in some trials [174,276,281,282] may not have been optimal. In contrast, menaquinones are more readily distributed to peripheral tissues [35,39,41], suggesting that lower doses may be sufficient to engage extrahepatic mechanisms. 

Taken together, the discrepancy between observational studies and RCTs in VK research should not be interpreted as evidence against biological relevance, but rather as a reflection of complex nutritional physiology combined with suboptimal trial design. Addressing baseline VK status, treatment duration, sex-specific effects, and the dose and form of supplementation will be essential to further determine whether VK plays a causal role in endocrine and cardiometabolic health.

### 8.4. Practical Implications and Future Directions

The findings presented here highlight several priorities for future research. As responses to VK supplementation appear to depend on baseline status, clinical studies should account for baseline VK status or preferentially target populations in whom extrahepatic VK insufficiency is most likely. Trials should also employ supplementation durations and doses sufficient to engage extrahepatic VK pathways. In studies on glucose metabolism, dynamic indices of insulin sensitivity should be incorporated to better characterize VK’s effects on peripheral insulin resistance. In addition, VK-dependent regulation of testicular testosterone synthesis is well supported experimentally but remains poorly defined in humans, representing an important research gap—particularly given the increasing prevalence of male hypogonadism associated with aging and obesity [283,284,285].

Beyond clinical trials, these findings also point to the potential public health relevance of VK insufficiency. Current dietary reference intakes for VK are based solely on coagulation outcomes and do not necessarily reflect requirements for the extrahepatic pathways involved in glucose and calcium metabolism. Furthermore, VK status shows marked interindividual variability that is only partly explained by dietary intake [86]. Apparent dietary adequacy may therefore coexist with functional insufficiency, and this state likely affects a substantial proportion of the population [9,10,11,12,16]. Together, these considerations highlight the plausible population-level relevance of functional VK status as a potentially modifiable factor in health conditions associated with impaired insulin sensitivity, skeletal fragility, and vascular calcification, all of which are increasingly prevalent worldwide [286,287,288].

### 8.5. Strengths and Limitations

The main limitation of this work is its narrative design. Unlike systematic reviews, it does not rely on a predefined search strategy or formal quality assessment, and selection bias cannot be excluded. In addition, the emphasis placed on individual studies may not fully reflect their methodological quality or statistical power. To address these limitations, this review distinguishes between study types, interpreting findings in light of their methodological context. Where available, greater emphasis is placed on meta-analyses and RCTs, while observational and mechanistic studies are used primarily to support biological plausibility and generate hypotheses. Accordingly, the interpretations presented should be viewed as context-dependent and hypothesis-generating rather than as definitive assessments.

Notwithstanding this limitation, this review has several strengths. First, it integrates mechanistic, observational, and interventional evidence to provide a comprehensive and up-to-date synthesis of VK’s effects on glucose metabolism—a field that has expanded rapidly in recent years. Second, it explicitly addresses key biological and methodological factors that complicate the interpretation of clinical evidence, including baseline VK status and ceiling effect, outcome selection, and heterogeneity in study populations. By doing so, the review goes beyond a descriptive summary of reported findings and offers a conceptual framework for understanding inconsistencies in the literature and for informing the design of future studies better suited to test VK-dependent mechanisms. Finally, by adopting a broad endocrine perspective, this review integrates evidence across multiple hormonal axes, and highlights emerging data supporting a plausible role for VK in male reproductive endocrinology.

## 9. Conclusions

Beyond coagulation, VK is increasingly recognized as a contributor to metabolic and endocrine processes. The mechanistic and clinical evidence reviewed here supports roles for VK in glucose metabolism and calcium homeostasis. In these domains, VK interacts with key hormonal pathways, including insulin signaling, PTH, and calcitriol. Clinically, VK supplementation has been associated with improvements in glucose metabolism, primarily in metabolically impaired populations, and appears to act synergistically with calcitriol, with combined interventions showing more consistent benefits in skeletal outcomes than either vitamin alone. The evidence for VK involvement in other endocrine axes remains preliminary and largely mechanistic. Together, these findings highlight the need for further well-designed studies that link mechanistic hypotheses to relevant clinical outcomes in order to clarify the endocrine significance of VK.

## Data Availability

Data sharing is not applicable to this article, as no datasets were generated or analyzed during the current study.

## Supplementary Materials

The following supporting information can be downloaded at https://www.mdpi.com/article/10.3390/nu18081183/s1, Supplementary Figure S1: The vitamin K cycle and vitamin K-mediated regulation of oxidative stress and inflammation; and Supplementary Table S1: Characteristics, interpretive value, and limitations of commonly used vitamin K status measures. References [25,36,66,74,75,91,121,122,123,124,125,126,127,128,129,130,131,132,133,134,135,136,137,138,139] are cited in the Supplementary Materials.
