# Supplementary material for: Vitamin K as an Endocrine Modulator: Mechanistic Links to Glucose Metabolism and Beyond
Source: Nutrients. 2026 Apr 9;18(8):1183. doi: 10.3390/nu18081183 (PMC13118554; doi:10.3390/nu18081183)
Supplement: Supplementary file 1 [file nutrients-18-01183-s001.zip › nutrients-4215524-supplementary.pdf]

Supplementary Materials

Vitamin K as an Endocrine Modulator: Mechanistic Links to Glucose Metabolism and Beyond

Wojciech Matuszewski [1,*], Mikołaj Madeksza [1], Michał Szklarz [1], Aleksandra Rutkiewicz [1], Joanna Rutkowska [1] and Joanna Maria Harazny [2,3]

1 Clinic of Endocrinology and Metabolic Diseases, School of Medicine, Collegium Medicum, University of Warmia and Mazury in Olsztyn, 10-957 Olsztyn, Poland; mikolaj.madeksza@icloud.com (M.M.); ola.rutkiewicz04@gmail.com (A.R.); michszklarz@gmail.com (M.S.); rutkowskaj14@gmail.com (J.R.)

2 Department of Pathophysiology, University of Warmia and Mazury in Olsztyn, Poland; joanna.harazna@uwm.edu.pl

3 Department of Nephrology and Hypertension, University Hospital Erlangen, Friedrich Alexander University Erlangen Nuremberg (FAU), 91054 Erlangen, Germany

* Correspondence: wmatuszewski82@wp.pl

Figure S1. The vitamin K cycle and vitamin K-mediated regulation of oxidative stress and inflammation.

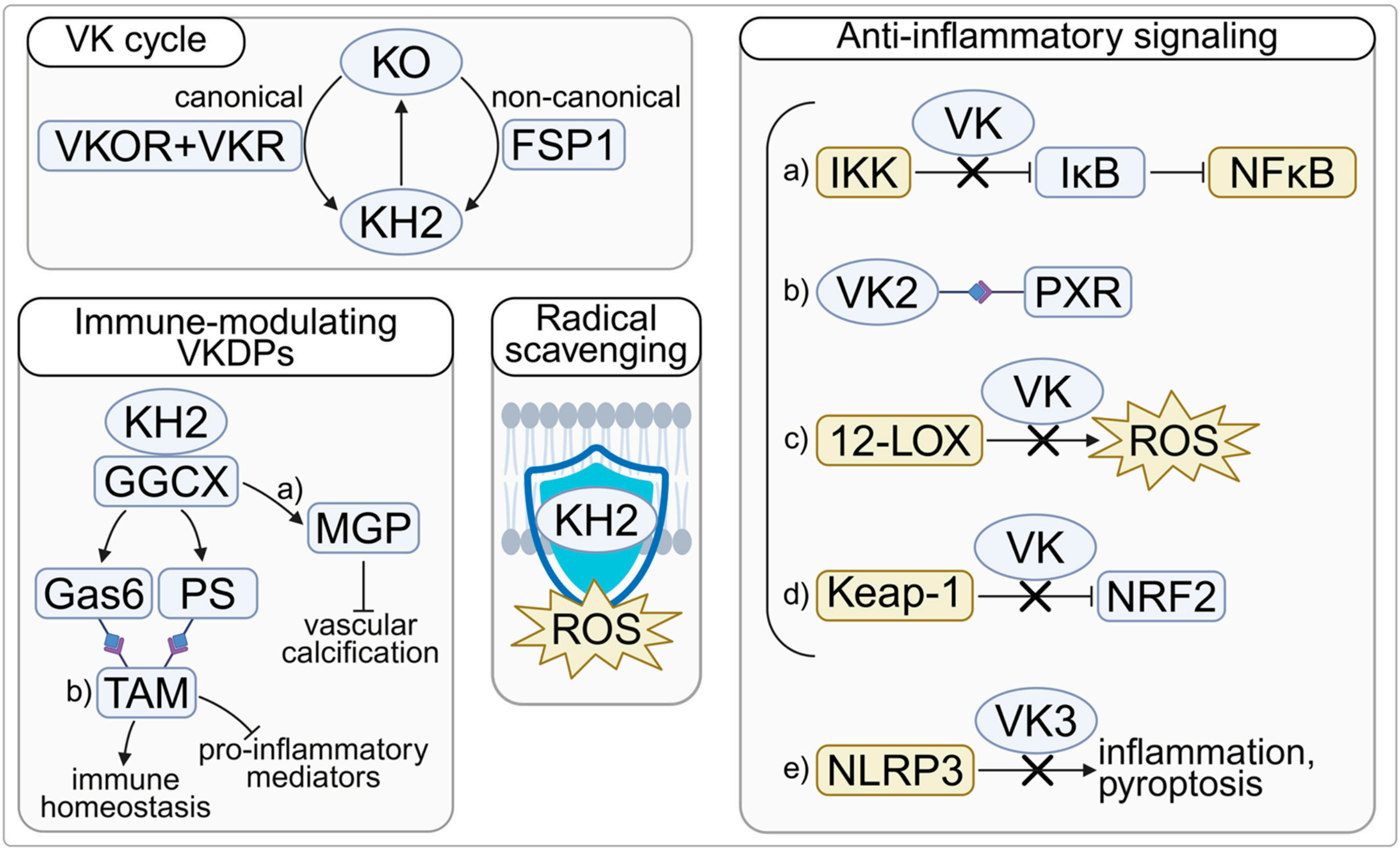

Notes: 1) Acting as a GGCX coenzyme, and a radical scavenger, KH2 is oxidized to KO, necessitating recycling through the VK cycle. In the canonical pathway, KO is reduced to VK by VKOR (or its paralog, VKORL1), and subsequently to KH2 by VKR [36,91,126]. In the non-canonical pathway, FSP-1 reduces KO directly to KH2 [25,74]; this pathway is primarily linked to KH2's antioxidant function [25]. 2) Immune-modulating VKDPs include MGP, Gas6, and Protein S. MGP inhibits inflammation-associated vascular calcification [122–125], while Gas6 and PS activate TAM receptors to promote immune homeostasis [127–132]. 3) KH2 acts as a radical-trapping antioxidant suppresses lipid peroxidation-driven ferroptosis [25,74,75]. 4) VK modulates inflammatory signaling through several signaling pathways, including: a) inhibition of NF-κB activation [121,133–135]; b) activation of PXR [66]; c) inhibition of 12-LOX activity in selected cell types [136]; d) activation of NRF2-dependent antioxidant responses via Keap-1 inhibition [121,137,138]; e) suppression of NLRP3 inflammasome activation [139]. Pathways a), b), and c) operate independently of the VK cycle [66,133,136].

Created in BioRender. Madeksza, M. (2026) https://BioRender.com/nc74xxi. Abbreviations: 12-LOX, 12-lipooxygenase; FSP1, ferroptosis suppressor protein 1; Gas6, growth arrest-specific protein 6; GGCX, γ-glutamyl carboxylase; IKK, IκB kinase; IκB, inhibitor of NFκB; Keap-1, Kelch-like ECH-associated protein 1; KH2, vitamin K hydroquinone; KO, vitamin K 2,3-epoxide; NFκB, nuclear factor kappa-light-chain-enhancer of activated B cells; NLRP3, NOD-, LRR- and pyrin domain-containing protein 3; NRF2, nuclear factor erythroid 2-related factor 2; PS, protein S; PXR, pregnane X receptor; ROS, reactive oxygen species; TAM, Tyro3-Axl-Mer receptor family; VK, vitamin K; VKDP, vitamin K–dependent protein; VKOR, vitamin K epoxide reductase; VKORL1, vitamin K epoxide reductase-like 1; VKR, vitamin K reductase.

Table S1. Characteristics, interpretive value, and limitations of commonly used vitamin K status measures

Measure	What it reflects	Strengths/typical use	Limitations
Dietary intake (food frequency questionnaire or similar dietary questionnaires)	Habitual VK intake (predominantly VK1; limited VK2 capture depending on database)	- Feasible and cost-effective for large epidemiological studies,<br>- Useful for ranking individuals by intake	- Subject to recall and reporting bias,<br>- Limited ability to estimate absolute intake,<br>- Confounded by overall diet quality and lifestyle (VK1 intake often reflects consumption of leafy greens and vegetable oils),<br>- May not accurately capture VK1 intakes >200 μg/day
Circulating phylloquinone (VK1)	Recent VK1 intake	- Objective biomarker,<br>- Responsive to dietary intake and supplementation	- Reflects short-term exposure (postprandial peak at ~6–10 h),<br>- Strongly influenced by triglyceride levels; fasting samples and lipid adjustment required,<br>- Inter-assay variability limits comparability across studies
Undercarboxylated osteocalcin (ucOC or %ucOC)	Functional VK availability in bone	- Sensitive marker of extrahepatic VK status,<br>- Responsive to dietary intake and supplementation	- Strongly influenced by bone turnover and total osteocalcin,<br>- Absolute ucOC concentrations are difficult to interpret unless expressed relative to total OC (%ucOC),
Circulating dp-ucMGP	Functional VK availability in vascular smooth muscle tissue	- Sensitive marker of extrahepatic VK status,<br>- Responsive to dietary intake and supplementation	- Influenced by age, vascular pathology, and total MGP levels,<br>- Associations may reflect disease burden rather than VK status per se
Circulating menaquinones (VK2)	Recent intake of specific menaquinones	- Potentially informative in populations with high MK intake (e.g. natto consumption, supplementation)	- Frequently undetectable in circulation at habitual intakes,<br>- Limited utility in most population-based studies,<br>- Limited validation against clinical outcomes
Circulating PIVKA-II	Hepatic VK sufficiency	- Clinically relevant in severe VK deficiency or anticoagulant use	- Low sensitivity within physiological VK intake ranges,<br>- Primarily reflects hepatic VK status, not extrahepatic tissues
Coagulation-based assays (PT/INR)	Activity of VK-dependent coagulation factors	- Widely available clinical tests	- Very insensitive to subclinical VK insufficiency,<br>- Not informative for nutritional VK status in healthy populations
Urinary metabolites [a]	VK turnover and metabolism	- Objective biochemical measures,	- Ideally require 24-h urine collection<br>- Rarely feasible in large studies<br>- Limited validation against clinical outcomes

[a] urinary biomarkers of VK metabolism include γ-carboxylglutamyl (Gla) acid – an indicator of the turnover of all VKDPs, and menadione – a VK metabolite. Notes: All currently available VK status measures respond to changes in intake over days to weeks; none reliably reflect long-term VK status independent of recent exposure. This table is provided to illustrate the evidence discussed in the manuscript; it does not represent a systematic search or formal quality assessment. Abbreviations: dp-ucMGP, dephosphorylated-undercarboxylated matrix Gla protein; INR, international normalized ratio; PIVKA-II, protein induced in vitamin K absence or antagonism-factor II; PT, prothrombin time; ucOCN, undercarboxylated osteocalcin; VK, vitamin K.
